# A genomic-clinicopathologic nomogram for predicting overall survival of hepatocellular carcinoma

**DOI:** 10.1186/s12885-020-07688-2

**Published:** 2020-12-01

**Authors:** Kena Zhou, Qiang Zhou, Congbo Cai

**Affiliations:** 1 Gastroenterology Department of Ningbo No.9 Hospital, Ningbo, 315000 Zhejiang China; 2 Emergency Department of Yinzhou No.2 Hospital, Ningbo, 315000 Zhejiang China

**Keywords:** Hepatocellular carcinoma, Overall survival, ICGC, TCGA, Nomogram

## Abstract

**Background:**

Hepatocellular carcinoma (HCC) is a common digestive tumor with great heterogeneity and different overall survival (OS) time, causing stern problems for selecting optimal treatment. Here we aim to establish a nomogram to predict the OS in HCC patients.

**Methods:**

International Cancer Genome Consortium (ICGC) database was searched for the target information in our study. Lasso regression, univariate and multivariate cox analysis were applied during the analysis process. And a nomogram integrating model scoring and clinical characteristic was drawn.

**Results:**

Six mRNAs were screened out by Lasso regression to make a model for predicting the OS of HCC patients. And this model was proved to be an independent prognostic model predicting OS in HCC patients. The area under the ROC curve (AUC) of this model was 0.803. TCGA database validated the significant value of this 6-mRNA model. Eventually a nomogram including 6-mRNA risk score, gender, age, tumor stage and prior malignancy was set up to predict the OS in HCC patients.

**Conclusions:**

We established an independent prognostic model of predicting OS for 1–3 years in HCC patients, which is available to all populations. And we developed a nomogram on the basis of this model, which could be of great help to precisely individual treatment measures.

## Background

Liver cancer is a deadly disease ranking the fourth leading cause of cancer related death. Hepatocellular carcinoma (HCC) accounts for 75–85% of liver cancer and is the main type for dead cases in liver cancer [1]. In the past decades, the evaluation of prognosis in HCC patients was usually based on the pathological diagnosis and clinical risk factors, such as AFP, DM, hypertension, hyperlipidemia, drinking, obesity and smoking [2–5]. However, the exact prediction ability of the above risk factors has never been described in previous articles.

With the widespread application of new generation RNA sequencing technology, more novel genes have been discovered playing important roles in tumors [6, 7]. For example, BIRC3 induces growth and metastasis both in vitro and vivo in HCC [8]; CTHRC1 is related to invasion and metastasis in liver cancer [9]; the expression level of OCIAD2 is related to growth and invasion in HCC [10]. Many literatures indicated that considering the low expression level of single gene, a combination of multiple genes can better predict the OS of HCC patients [11, 12]. However, such models cannot accurately predict the OS of HCC patients, which has an impact on making decision about clinical treatment [13]. A nomogram can solve this problem very well, because it can accurately predict the OS of HCC patients based on the exact weighted score [14].

We developed a 6-mRNA prognostic model based on the ICGC database, which was verified in the TCGA database. And this model can independently predict the OS of HCC patients. Moreover, we used the 6-mRNA risk score and related clinical risk factors to draw a nomogram, which can accurately assess the OS of HCC patients for 1–3 years.

## Methods

### Data source

**Fig. 1:**
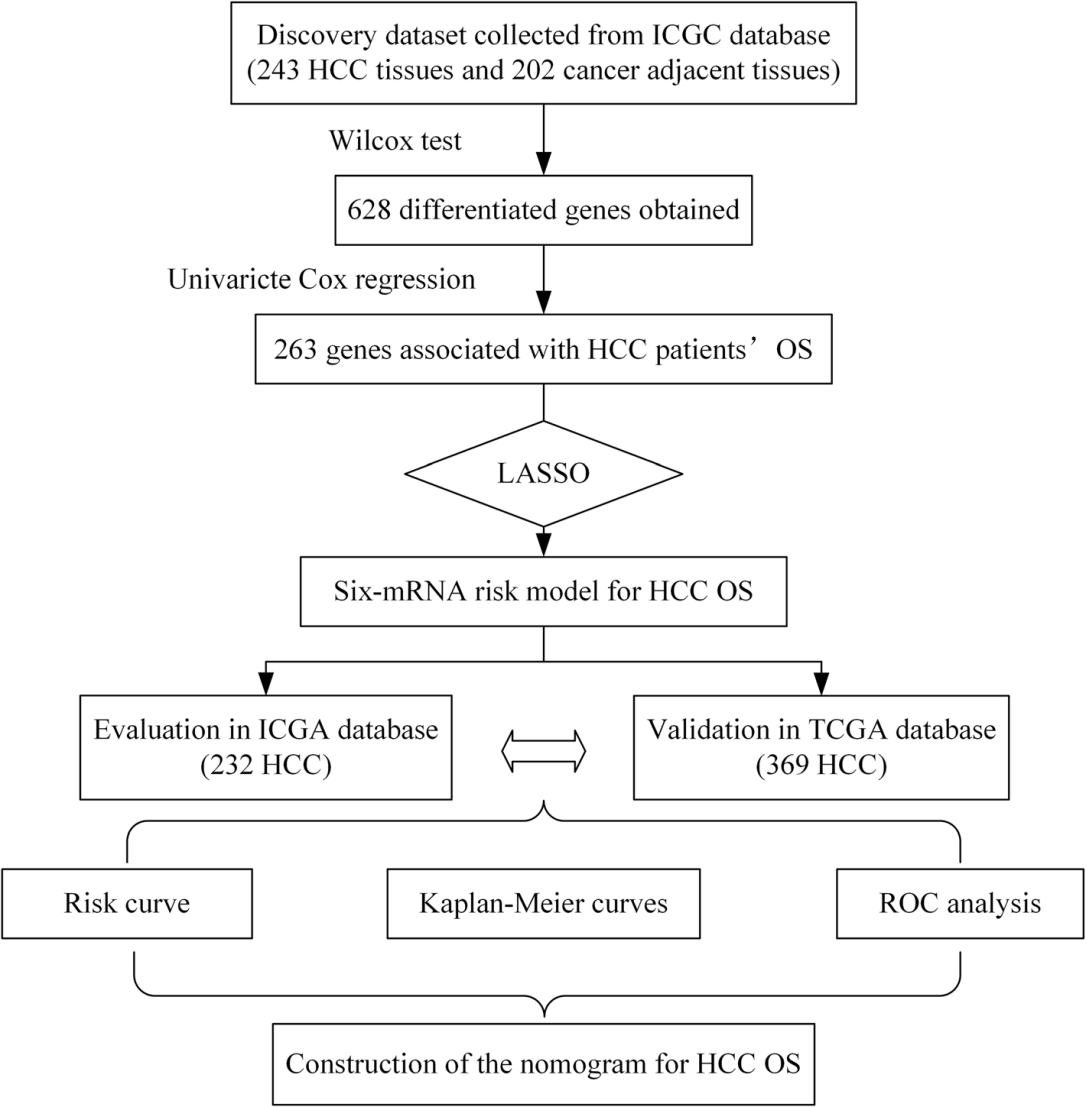
The flow chart of bioinformatics analysis

The experimental group data was downloaded from the ICGC data portal (https://icgc.org/) until July 7, 2019. It included the RNA-Seq expression and corresponding clinical information of 243 liver cancer samples and 202 cancer adjacent tissues. The validation group data was gained from the TCGA data portal (https://cancergenome.nih.gov/) until July 29, 2019. It contained the RNA-Seq expression and clinical characteristics of 374 HCC tumor specimens and 50 cancer adjacent tissues. Both ICGC and TCGA data are publicly available; therefore, approval from the local ethics committee is not needed. The research flow chart is shown in Fig. 1.

### Screening of differential genes

RNA-Seq data of HCC samples and cancer adjacent samples were compared using the Wilcox test in the R language to identify genes with differential expression. |Log fold change (FC)| > 2 and false discovery rate (FDR) < 0.05 is regarded as the threshold for the differential gene, and differentially expressed genes were screened out for subsequent analysis. Volcano plots were performed using Prism 6.0 software.

### Forming up of the prognostic model

We performed univariate cox analysis with the “survival” R package on each differential gene to identify their association with OS. Genes with *P* < 0.05 were considered to be related to the OS of HCC, and were selected for further analysis. Due to the large number of genes, we performed a LASSO regression analysis using the “glmnet” R software package. Finally, the best prognostic model is established based on the minimum value of cross-validation error. Patients were divided into high- and low-expression groups, based on their mRNA expression levels. Survival curves for each mRNA were generated using “survival” R package.

### Evaluation of the prognostic model

Multivariate cox regression was conducted to calculate the risk value of each patient according to the established model. The formula of risk value is as follows: $\mathrm{Risk}\ \mathrm{score}={\sum}_{\mathfrak{i}=1}^{\mathrm{N}}\left(\mathrm{Ei}\ast \mathrm{Ci}\right)$, where N is the number of prognostic genes and Ci is the coefficient of the i-th gene in the multivariate Cox regression analysis; Ei is the expression value of the i-th gene. Ci > 0 is defined as a high-risk gene, and Ci < 0 is defined as a protective gene. Then a concise forest map for the model was drafted based on multiple regression analysis.

All 232 patients were ranked according to the risk value, and the scores were divided into two groups according to the median (high risk and low risk). A heat map was drawn using R package “pheatmap” for the comparison of 6 mRNAs’ expression in high-risk and low-risk groups.

Risk survival curves were plotted using the Kaplan-Meier method and the time difference in OS between the two groups was compared using a two-sided rank sum test. The sensitivity and specificity of the prognostic model prediction were assessed by the area under the curve (AUC) of the ROC, applying the R package “survival ROC”. The model’s consistency test (C-index) was got using the R package “survcomp” to verify the reliability.

Three hundred sixty-nine HCC patients’ characteristics was downloaded from TCGA database as a verification group, and we calculated their risk values. Then the K-M curve is drawn to verify the applicability of the model in the TCGA database.

### A nomogram to predict OS of liver cancer patients

Univariate and multivariate cox regression analyses were applied on traditional clinical factors (age, gender, stage, and previous tumors) on the 6-mRNA prognostic model to determine that whether the model was affected by conventional clinical factors. The R package “rms” was used to establish a composite nomogram for all risk factors of the OS. Each risk factor in the nomogram has a specific score, and their sum represents the total score of the HCC patient. This is used to predict the OS of HCC patients in the near 1 to 3 years.

## Results

### Patient characteristics

The experimental members in this study were 232 Japanese liver cancer patients from the ICGC database, among which 192 HCC patients had adjacent tissue matching, 31patients had HCC samples, and 9 patients had multiple cancer tissues or adjacent tissues. In total 243 HCC samples and 202 cancer adjacent samples were included. The validation group contained 369 HCC patients from the TCGA database, and their clinical and pathological characteristic is detailed in Table 1. In the experimental group, we compared 234 tumor cases and 202 normal cases by direct sequencing to find differential genes. Appendix Table 1 shows that there is no significant difference between normal samples and tumor samples from the ICGC on the aspects of age, gender, vital status, tumor stage and prior malignancy (*p* > 0.05).

**Table 1:** Summary clinical characteristics of included patients

Category	Experimental group (ICGC, *n* = 232)	Validation group (TCGA, *n* = 369)
Age		
< 65	83 (35.8%)	218 (59.1%)
≥ 65	149 (64.2%)	151 (40.9%)
Gender		
Male	171 (73.7%)	248 (67.2%)
Female	61 (26.3%)	121 (32.8%)
Vital status		
Alive	189 (81.5%)	243 (65.9%)
Dead	43 (18.5%)	126 (34.1%)
Race		
White	0	185 (50.1%)
Black	0	17 (4.6%)
Asian	232 (100%)	157 (42.6%)
unknown	0	10 (2.7%)
Tumor stage		
I	36 (15.5%)	172 (46.6%)
II	106 (45.7%)	84 (22.8%)
III	71 (30.6%)	85 (23.0%)
IV/	19 (8.2%)	5 (1.4%)
unknown	0	23 (6.2%)
T stage		
T1	NA	181 (49.1%)
T2	NA	92 (24.9%)
T3	NA	80 (21.7%)
T4	NA	13 (3.5%)
unknown	NA	3 (0.8%)
M stage		
M0	NA	266 (72.1%)
M1	NA	4 (1.1%)
unknown	NA	99 (26.8%)
N stage		
N0	NA	251 (68.0%)
N1	NA	4 (1.1%)
unknown	NA	114 (30.9%)
Prior malignancy		
Yes	30 (12.9%)	NA
No	202 (87.1%)	

NA: Clinical data are unknown

### Differentially expressed mRNA between HCC and cancer adjacent tissues

**Fig. 2:**
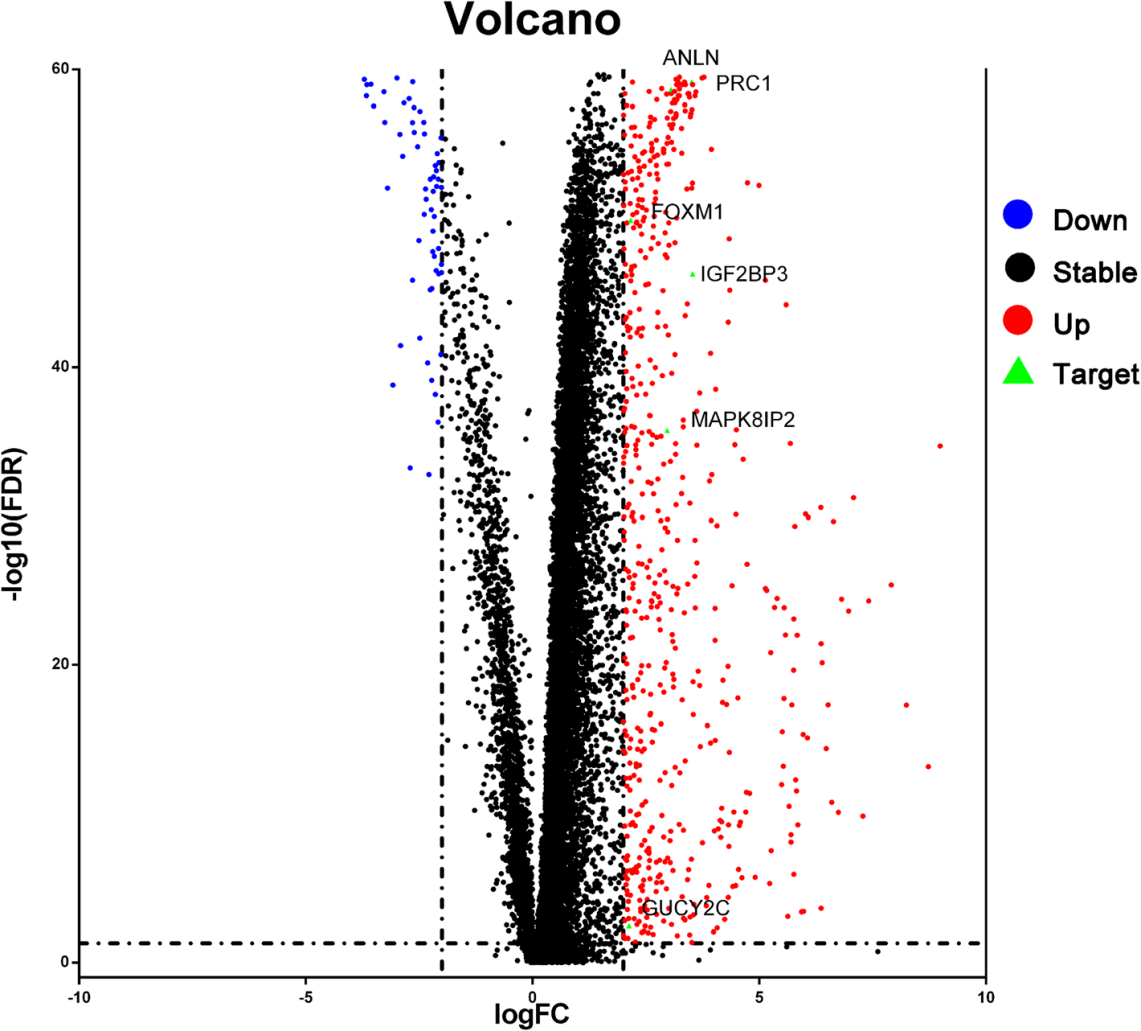
Volcano plot of differentially expressed miRNAs. Blue dots represent downregulated mRNAs, Black dots represent stable mRNAs, Red dots represent upregulated mRNAs, AND Green triangles represent the target 6 mRNAs

The RNA-Seq expression profile of liver cancer patients from the ICGC database covers a total of 243 tumor tissues and 202 cancer adjacent tissues. Wilcox test was conducted in R language to compare the genetic differences between HCC samples and cancer adjacent samples. And totally 628 genes with differential expression were filtered out. The 569 upregulated and 59 downregulated mRNAs are provided in a volcano plot (Fig. 2).

### Set up of the prognostic model

**Fig. 3:**
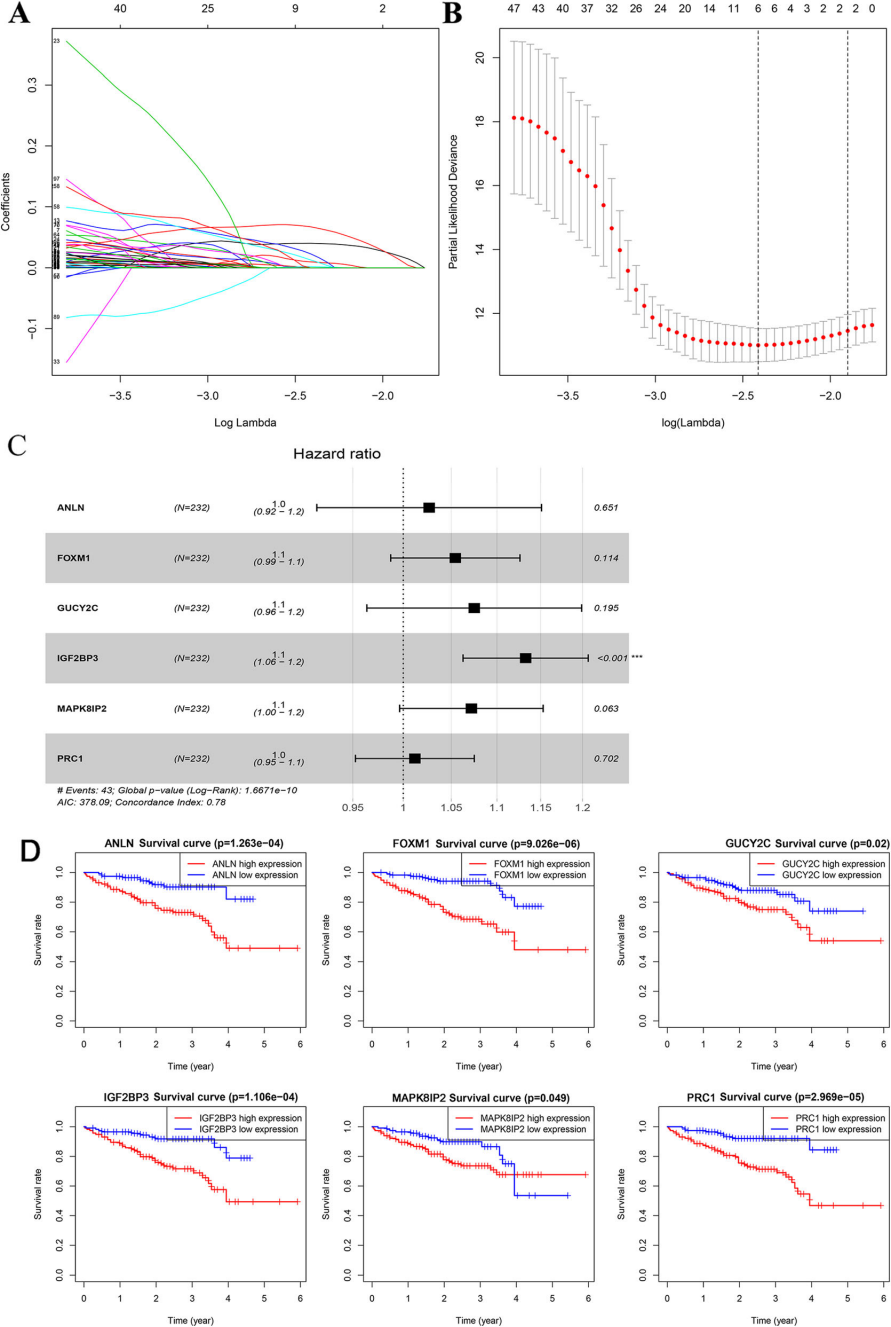
the OS related mRNA extraction and model establishment. **a** LASSO regression coefficient profile of 263 survival-related mRNAs. **b** “Leave-one-out-cross-validation” for parameter selection in LASSO regression. **c** Forest plot for the association between 6-mRNA and risk value. **d** Kaplan-Meier curves for 6 mRNAs

Two hundred sixty-three of 628 differential genes were discovered to be closely related to OS in HCC patients (*P* < 0.05) using the univariate Cox regression. Afterwards, 1000 LASSO Cox regression analyses were performed on the above 263 genes to select the most informative genes (Fig. 3a). And means of “leave-one-out-cross-validation” was utilized to pick the target model (Fig. 3b). Hazard ratios of six mRNAs are demonstrated in the forest plot. All these 6 mRNAs’ expression levels are regarded to be risk factors for HCC because of Coef > 0. The overall *P* value is 1.6671E-10 and the model has a C-index of 0.78 (Fig. 3c). The K-M curve illustrated that survival rate decreased over time. Higher mRNA expression indicates lower survival rate in HCC patients (Fig. 3d). And the differences were considered statistically significant at *P* < 0 .05. Although it seems that the survival rate of higher expression group of MAPK8IP2 was higher than the lower expression group after year 4. We found that this phenomenon was due to a sudden decrease in the follow-up population. In summary the mortality rate was reduced when MAPK8IP2 was lower expression (*P* < 0.05).

A prognostic model of 6-mRNA was set up through LASSO regression analysis (Table 2). The linear combination of the expression values of the six mRNAs weighted by the multivariate cox regression coefficients, the risk score formula is as follows: (0.0264× expression value of ANLN) + (0.0530× expression value of FOXM1) + (0.0722× expression value of GUCY2C) + (0.1245× expression value of IGF2BP3) + (0.0693× expression value of MAPK8IP2) + (0.0118× expression value of PRC1).

**Table 2:** 6 mRNAs screened out by LASSO regression analysis

Gene	Coef	HR	HR (95%CI)	Pr (>|z|)
ANLN	0.0264	1.0267	0.9158–1.1510	0.651256
FOXM1	0.0530	1.0544	0.9873–1.1260	0.114215
GUCY2C	0.0722	1.0749	0.9636–1.1990	0.19508
IGF2BP3	0.1245	1.1326	1.0627–1.2070	0.000126*
MAPK8IP2	0.0693	1.0718	0.9963–1.1529	0.062675
PRC1	0.0118	1.0119	0.9525–1.0749	0.70183

*Coef* Coefficient; *: *P* < 0.01; *HR* Hazard ratio

### Assessment and validation of the prognostic model

**Fig. 4:**
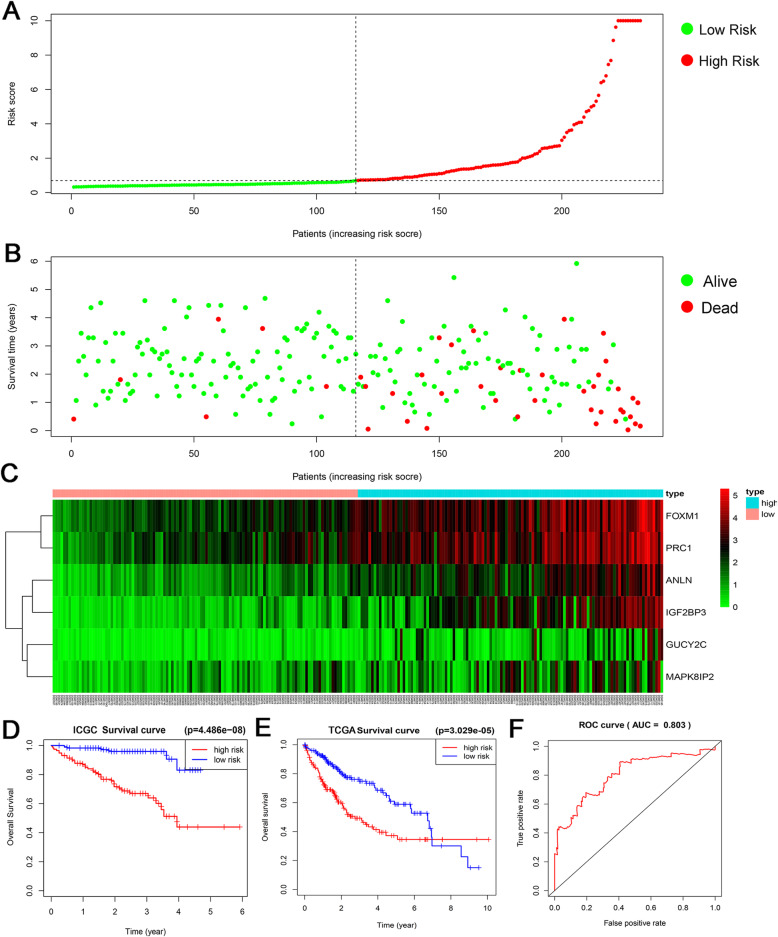
Evaluation and validation on the 6-mRNA prognostic model. **a** mRNA risk score distribution, **b** Survival status and survival time of HCC patients, **c** Expression profiles of 6 mRNAs in 232 patients. **d** Kaplan-Meier curve of OS between the high-risk group and the low-risk group in ICGC database. **e** Kaplan-Meier curve of OS between the high-risk group and the low-risk group in TCGA database. **f** ROC curve of 3-year survival rate, the AUC value of the area under the ROC curve is 0.803

The risk value of 232 HCC patients in the ICGC database was calculated applying the above-mentioned risk score formula. Then we ranked the risk values for all patients and divided them into a high-risk group (116 patients) and a low-risk group (116 patients) based on the cut-off value of 0.6943 (Fig. 4a). We found that HCC patients with increasingly higher risk value tended to have shorter survival time and lower survival rate (Fig. 4b). In addition, the expression value of the 6-mRNA increased simultaneously with the risk score, which could be clearly seen in the heat map (Fig. 4c).

The Kaplan-Meier curve indicated that the low-risk group was likely to hold a longer survival time (*P* < 0.001). However, a significant reduction was found in follow-up after 3 years and a lack of follow-up population after 5 years (Fig. 4d).

To further confirm the applicability of the 6-mRNA prognostic model, we verified it in the TCGA database. We used this prognostic model to calculate the risk value of 369 patients in the TCGA database. A Kaplan-Meier survival curve showed that the survival rate of the high-risk group was significantly lower than that of the low-risk group in the early 7 years. Due to the rapid decrease in follow-up, the survival rate of the high-risk group was higher than that of the low-risk group after 7 years. Anyhow, from the overall assessment of TCGA’s 10-year follow-up data, the risk of death in the high-risk group was significantly higher than that in the low-risk group (*P* = 3.029E-05) (Fig. 4e).

The area under the ROC curve of the 6-mRNA prognosis model is 0.803, indicating that the established model has high specificity and sensitivity in predicting OS in HCC patients (Fig. 4f). The C index value is 0.7797 (95% CI: 69.5–86.5%, *P* value: 1.1543E-10), further proving the high accuracy of the model.

### Independence test of prognosis model and development of nomogram

In order to investigate whether the 6-mRNA prognostic model can be an independent prognostic factor for predicting OS in HCC patients, we conducted the following tests. Firstly, univariate cox analysis was applied, and we found that the 6-mRNA prognostic model and the stage were significantly associated with OS in patients with HCC (*P* < 0.01). Secondly, multivariate cox analysis showed that the 6-mRNA prognostic model was independent from other clinical factors and could be used as an independent prognostic factor for predicting OS in HCC patients (HR = 1.1031, *P* = 3.00E-11) (Table 3).

**Table 3:** Independent predictive power of the 6-mRNA model in HCC patients

Variables	Univariate analysis			Multivariate analysis		
	HR	HR (95% CI)	*P* value	HR	HR (95% CI)	*P* value
6-mRNA risk Score	1.1109	1.0837–1.1388	1.03E-16***	1.1031	1.0717–1.1355	3.00E-11***
Age	1.0200	0.9719–1.0330	0.8985	1.0054	0.9716–1.0405	0.7558
Sex (Male/ Female)	0.5185	0.2782–0.9664	0.0387*	0.3381	0.1742–0.6564	0.0014*
Stage (G1/ G2/ G3/ G4)	2.1546	1.4929–3.1098	4.13E-05***	1.9287	1.2998–2.8619	0.0011*
Prior Malignancy (No/ Yes)	1.7510	0.7733–3.9648	0.1791	2.5483	1.0741–6.0459	0.0338*

*: *P* < 0.05; **: *P* < 0.01; ***: *P* < 0.001

**Fig. 5:**
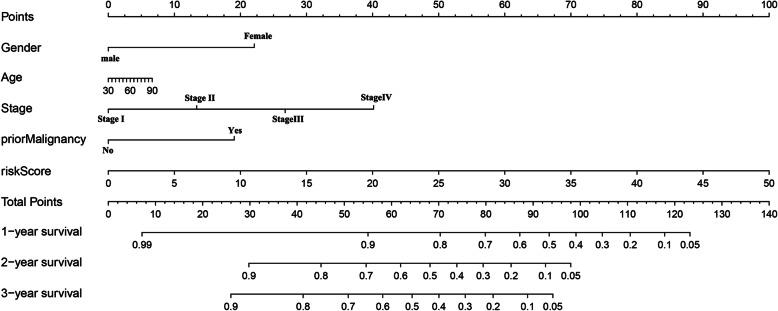
The composite nomogram consists of the six-mRNA score, gender, age, stage, and prior malignancy score

We have produced a nomogram of various risk factors. And scores were calculated according to various risk factors’ *P* values after the multi-factor Cox analysis. The smaller the *P* value is, the higher the score of the influencing factor in the nomogram is. Thus, the specific score values of the 6-mRNA risk score and traditional clinical data can be obtained separately. Adding up all the values to calculate the total score, a vertical line in the nomogram could be obtained to get an accurate OS for HCC patient for 1–3 years (Fig. 5).

## Discussion

HCC is a fatal malignant tumor with high heterogeneity, and it is difficult to obtain an accurate OS, which affects the clinical decision-making and treatment of HCC patients [15]. We obtained the 6-mRNA prognosis model through the ICGC database, which is also applicable in the TCGA database. And this model can independently evaluate the OS of HCC patients. Moreover, we used 6-mRNA score, gender, age, stage, and prior malignancy score to develop a nomogram for accurately assessing the OS of HCC patients.

Pathological results and AFP levels are often used clinically to predict the prognosis of HCC patients, but they cannot evaluate the OS in HCC patients exactly [16]. With the development of molecular biology, many new biomarkers have emerged to prompt the prognosis of HCC patients. Peptide SMIM30 promotes HCC development by inducing SRC/YES1 membrane anchoring and MAPK pathway activation [17]. Ectosomal PKM2 promotes HCC by inducing macrophage differentiation and remodeling the tumor microenvironment [18]. And the prognostic model composed of multiple genes can better predict the OS in HCC patients [13]. Zhou T et al. found a novel ten-gene signature that could predict prognosis in HCC [19]. Here we selected out 6 mRNAs via bioinformatics, and previous studies suggest that their potential functions were as follows: ANLN was mainly expressed in the nucleus and showed significantly higher expression levels in cancerous tissues than those in paired adjacent tissues. Overexpression of ANLN is related to poor prognosis in patients with hepatocellular carcinoma [20]. Inhibiting ANLN in liver cells can prevent cell division and inhibit the development of liver tumors in mice. Drugs that inhibit ANLN in the liver may be effective in preventing or treating HCC. Knockdown of Anillin Actin Binding Protein Blocks Cytokinesis in Hepatocytes and Reduces Liver Tumor Development in Mice Without Affecting Regeneration [21]. FOXM1 promotes hepatocellular carcinoma progression by regulating KIF4A expression [22]. IGF2BP3 increases HCC cell invasiveness by promoting the miR191-5p-induced suppression of ZO-1 signaling [23]. We identified PRC1 as a novel Wnt target that functions in a positive feedback loop that reinforces Wnt signalling to promote early HCC recurrence [24]. Currently there’s no relevant literature on the mechanisms of MAPK8IP2 and GUCY2Cin HCC.

In this study, we used an external database to verify the 6-mRNA prognosis model to ensure the wider applicability. We think this process has important clinical significance. HCC specimens in the experimental group from the ICGC database were derived from Asian population, and the verification group from the TCGA database was mainly white and black. We validated the prognostic model of 6-mRNA through different platforms. It is proved that the model we established was applicable to all populations. Many studies have obtained prognostic models of multiple genes through the TCGA database, but the prognostic model cannot accurately predict the OS in HCC patients [25–27]. Hence, we further made a genomic-clinicopathologic nomogram containing the model as one factor to solve the accuracy problem.

We need to consider some imperfections in our study. Firstly, follow-up time for HCC patients in the ICGC database is relatively short, with a median of 27 months. Since most patients die within 3 years, the nomogram mainly predicts the OS in HCC for 1–3 years. Secondly, the ICGC database lacks relevant clinical information, such as smoking, drinking, and hepatitis, we cannot consider these factors. Since ICGC uses LCSGJ (Liver Cancer Research Society of Japan) staging system and TCGA uses AJCC (American Cancer Council) staging system, possible errors in verification process need to be considered although with our efforts to avoid their differences. Furthermore, the specific molecular biological mechanism and pathway of 6-mRNA are still unknown.

## Conclusion

Our study confirms a new prognostic model of 6-mRNA that can independently predict OS in HCC patients. And this model has been validated in another external database, which is available to all population. Furthermore, we plotted a genomic-clinicopathologic nomogram for predicting OS in HCC patients for 1–3 years, which is more accurate than TNM staging and other prognostic models. Definitely, our study could contribute to decision-making in the precision medicine of HCC patients.

## Supplementary Information

**Additional file 1 Appendix Table 1**: Summary of clinical characteristics in normal and tumor groups

## Data Availability

The datasets generated and analysed during the current study are available in the ICGC repository (https://icgc.org/) and TCGA repository (https://cancergenome.nih.gov/).

## Abbreviations

AFP: Alpha fetoprotein
AUC: Area under the ROC curve
FC: Fold change
FDR: False discovery rate
HCC: Hepatocellular carcinoma
ICGC: International Cancer Genome Consortium
LASSO: Least absolute shrinkage and selection operator
OS: Overall survival
TCGA: The Cancer Genome Atlas
LASSO: Least absolute shrinkage and selection operator
LCSGJ: Liver Cancer Research Society of Japan
AJCC: American Cancer Council
