# Supplementary material for: A genomic-clinicopathologic nomogram for predicting overall survival of hepatocellular carcinoma
Source: BMC Cancer. 2020 Dec 1;20:1176. doi: 10.1186/s12885-020-07688-2 (PMC7709450; doi:10.1186/s12885-020-07688-2)
Supplement: Supplementary file 1 — Additional file 1 Appendix Table 1: Summary of clinical characteristics in normal and tumor groups [file 12885_2020_7688_MOESM1_ESM.docx]

**Appedix Table 1: Summary of clinical characteristics in normal and tumor groups**

| Category | Normal group  (n=202) | Tumor group  (n=243) | χ^2^ | P |
| --- | --- | --- | --- | --- |
| Age |  |  |  |  |
| <65  ≥65 | 74 (36.6%)  128 (63.4%) | 87 (35.8%)  156 (64.2%) | 0.033 | 0.856 |
| Gender |  |  |  |  |
| Male  Female | 145 (71.8%)  57 (28.2%) | 182 (74.9%)  61 (25.1%) | 0.549 | 0.459 |
| Vital status |  |  |  |  |
| Alive  Dead | 162 (80.2%)  40 (19.8%) | 199 (81.9%)  44 (18.1%) | 0.207 | 0.649 |
| Tumor stage |  |  |  |  |
| I  II  III  IV/ | 26 (12.9%)  93 (46.0%)  65 (32.2%)  18 (8.9%) | 36(14.8%)  110(45.3%)  76(31.3%)  21(8.6%) | 0.351 | 0.950 |
| Prior malignancy |  |  |  |  |
| Yes  No  unknown | 68 (33.7%)  124 (61.4%)  10 (4.9%) | 78 (32.1%)  150 (61.7%)  15 (6.2%) | 0.378 | 0.828 |

Unknown: Clinical data are unknown
